# Cleft Palate induced by Sulfur Mustard in mice fetus

**Published:** 2012

**Authors:** Mohammad Hassanzadeh-Nazarabadi, Nasrin Sanjarmoosavi, Naser Sanjarmoosavi, Sahar Shekouhi

**Affiliations:** 1 *Medical Genetics Department, Faculty of Medicine, Mashhad University of Medical Sciences, Mashhad, Iran*

**Keywords:** Sulfur Mustard, teratogenicity, cleft lip/palate

## Abstract

Sulfur Mustard (SM) is a chemical warfare agent which was widely used in the World War I and more recently during Gulf war in the early 1980s'. SM is a strong alkylating agent with known mutagenic and carcinogenic effects; but only few studies have been published on its teratogenicity. Since SM has been widely used as a chemical weapon by the Iraqi regime against the Iranian soldiers as well as the civilian population particularly pregnant women in the border area; therefore, the investigation of SM adverse effects on cleft malformations which is one of the most frequent congenital anomalies is considered in this study. An experimental work has been carried out in embryopathy in mouse with intraperitoneal injection of 0.75 and 1.5 mg/kg SM at different periods of gestation. Cleft lip and palate were examined by stereomicroscopy. Current data demonstrate that exposure with SM on the 11th day of gestation can increase the incidence of cleft defects in comparison with control group (P<0.001). These results also show that SM treatment in GD 11 and 13 can lead to more anomalies compared with GD 14 (P<0.001). They also show that the teratogenic effects of SM are restrictively under the influence of the threshold dose and time of gestation. The present results suggest that exposure to sufficient doses of SM on critical days of gestation may increase the risk of congenital cleft malformations.

In World War І (1914-1918), the use of chemical weapons especially mustard gas (SM) led to thousands of death (1,2,3). Without attention to the conventional laws that prohibit the use of these weapons, these agents were applied by the Iraqi Army during the Gulf war (1981-1989) which caused the deaths of many soldiers (1).

The destructive effects of SM are well recognized. The eyes, the skin, and the respiratory tract are the principal organ targets of SM toxicity (4-8). SM is highly lipophilic and is absorbed very quickly through the skin. After a latent period of 6-24 h erythema and blisters appear on the skin (6). Pulmonary complications mainly on the upper respiratory tract such as hemorrhagic inflammation, sore throat, hoarseness, cough, bronchitis, and bronchopneumonia are observed in SM-exposed victims (6,9). Additionally, lung cancers had been reported in fishermen who were exposed to SM and in workers of SM manufacturing plants (10-12). Because of its alkylating and electrophilic properties, SM can alter chemical functional groups such as amines, carboxyls, S-H and O-H groups, and also primary phosphate groups (4). There are three distinct biochemical effects of SM: cytostaticity, mutagenicity, and cytotoxicity (4). Although considerable work has been focused on understanding the mechanisms of direct cellular injury mediate by SM exposure, relatively little is known about this phenomena. Several mechanisms have been proposed for the cytotoxicity of SM including; DNA damage, labilization of lysosomes and calcium mediated toxicity (6,7,13,14). SM like other mustards agents such as nitrogen mustard may possess teratogenic effects (15).

Craniofacial malformations are major human birth defects (16). with a worldwide frequency of 1 in 700 and substantial clinical impacts (17-19). Facial clefts represent the majority of these defects and can rise at any stage of development due to perturbation that alter the extracellular matrix as well as affect the patterning, migration, proliferation, and differentiation of cells (16). These deformities are believed to be caused by multifactorial inheritance of a threshold characteristic where several genes interact with environmental agents (4,20,21). An environmental component to clefting was recognized when Warkany et al. associated nutritional deficiencies with cleft palate (17,22). In addition, clefts may vary according to several influencing factors including time (4,23-28) and race (4,29-31). This report minimizes the time and race variability factors, thus focusing more precisely on environment and in particular SM-exposure.

In the event of an SM attack during war or a terrorist incident, the pregnant women might be one of the victims who survive the SM-exposure. However, the transplacentally exposed fetus may bear long term consequences. 

Since comparatively little work has been conducted to assess the impact of SM on fetus teratogenicity, investigation of SM developmental toxicity should be considered. The aim of this study was to define the teratogenic effects of SM on cleft lip/palate on mouse embryo.

## Methods and Material

Reagents: Phenytoin (Dilantin®) was obtained from Parke Davis Company. SM (purity of 99.8%) was donated by Mashhad College of Pharmacy. Propylene Glycol was purchased from Merck Company (Germany). All other chemicals were of analytical grade and commercially available. All prepared solutions were stored at 4°C in the dark until administration.

Animals Care Statement: Both sexes of N. meri albino mice (mice south) were purchased from Razi Institute (Hesarak, Iran) and acclimatized for one week prior to treatment. Throughout the experiment, the mice were housed in a specific pathogen-free facility on corncob bedding with food and water ad libitum. The mice were randomly assigned to control and test groups. Seven mice were housed in each group.The gestational Day (GD) was defined as the date on which the vaginal plug was observed.

Animal Treatment: Pregnant females were IP dosed with 0.75 and 1.5mg of SM/kg of body weight. These doses were applied with regard to LD50 of 4.4mg/kg on GD 7; (32) the dose that will kill 50% of a group of animals under stated conditions. The control group was given the same volume of Phenytoin or Propylene Glycol. The schedule of administration is outlined in Table 1. On GD19, the mice were sacrificed by overdose of sodium thiopental. The gravid uterus of the pregnant mouse was harvested and weighed. The numbers and positions of the live or dead fetuses, as well as reabsorptions, were recorded. The live fetuses were weighed individually, gender determined and examined for external abnormalities.

**Fig 1:**
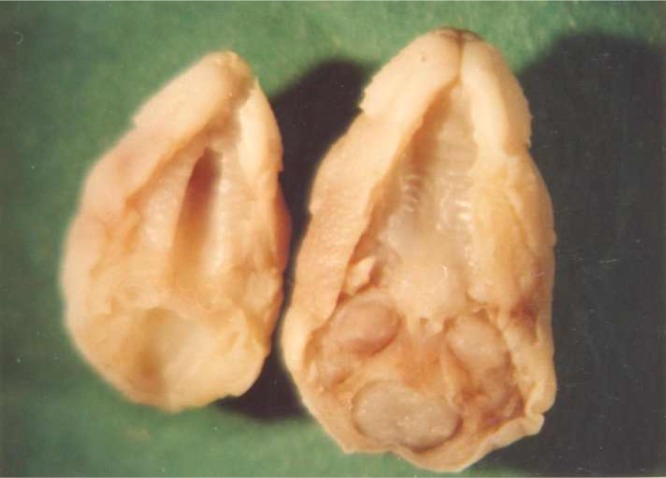
Cleft Palate under Stereomicroscopy (left) and normal palate (right

Normal palatogenesis was assessed based on microscopic examination of the palate surface after an incision was made through the temporal-mandibular joint. The cleft palate was scored if there was not fusion between the secondary palatal shelves (Fig.1).

These experiments were performed under the ethical guidance of Animal House of Ghaem Hospital, Mashhad University of Medical Sciences. Statistics: statistical analyses were plotted using Microsoft Excel. Data were analyzed by Chi-Square test followed by Fisher's Exact Test. The level of p<0.05 was considered significant.

**Table 1:** IP injection Schedule of different drugs with definition of fetuses and the frequency of anomalies

**No.**	**Used** **material**	**Number of** **pregnant** **mice**	**Day of** **injection**	**Injection** **dose**	**Injection** **volume**	**Live Fetus**	**Dead Fetus**	**Resorbed Fetus**	**Mean Fetal Weight**	**CP**
**1**	Phenytoin	9	G.D. 12	0.75 mg/kg	0.1 ml	77	0	0	1.33 ± 0.17	34
**2**	_	8	_	_	_	74	0	0	1.34 ± 0.18	0
**3**	Propylene Glycol	8	G.D. 11	1.5 mg/Kg	0.1 ml	66	0	0	1.33 ± 0.23	0
**4**	Propylene Glycol	7	G.D. 13	1.5 mg/Kg	0.1 ml	69	0	0	1.33 ± 0.24	0
**5**	SM	5	G.D. 11	1.5 mg/Kg	0.1 ml	50	3	9	0.85 ± 0.39	28
**6**	SM	6	G.D. 13	1.5 mg/Kg	0.1 ml	59	0	4	0.92 ± 0.5	21
**7**	SM	7	G.D. 14	1.5 mg/Kg	0.1 ml	55	0	0	1.1 ± 0.48	0
**8**	SM	6	G.D. 11	0.75 mg/Kg	0.1 ml	62	1	0	0.81 ± 0.13	12
**9**	SM	5	G.D. 13	0.75 mg/Kg	0.1 ml	46	0	0	1.33 ± 0.2	0

GD; Gestational Day, CP; Cleft Palate

## Results

The results of pregnancy in SM treated groups are compared with control groups in 9 groups (Table 1). No indicative organ anomalies were observed in control negative and solution control groups. These results show that the incidence of cleft malformations in Phenytoin treated group was higher than control groups. In addition, the current data demonstrate that injection of 1.5 mg/kg in GD 11 significantly increase the incidence of cleft anomalies in comparison with the control group (p<0.001), but no obvious teratogenic activity of SM could be observed on GD14. The rate of anomalies was also slightly higher in GD11 compared with GD13. On the other hand, the incidence of malformations were more prominent in the 1.5 mg/kg than 0.75 mg/kg (p=0.01).

## Discussion

Sulfur mustard (SM), commonly known as mustard gas, is an alkylating agent which was widely used as a chemical warfare during Gulf war against soldiers and civilians (1). The previous reports have demonstrated the ability of this class of compounds to cause adverse effects (15). However, very few correlations have been established between SM exposure and congenital cleft lip/palate deformity. Similar experimental works were carried out on its analog; Nitrogen Mustard, which revealed that it can lead to different malformations such as: cleft palate, functional and structural anomalies and some growth defects (15). These data demonstrate that the teratogenic effects of SM are restrictively under the influence of the gestation time (during organogenesis) and the threshold dose. The critical period of the different organs may interfere and therefore, exposure to a single teratogen in a specific day may cause several anomalies. On the other hand, the organ specific critical period may take several days long and the sensitivity of organs to teratogens can vary greatly in different periods. Therefore, a specific dose of a teratogen in different days may cause different anomalies and increases the rate of malformations. Teratogens can interfere with cleft morphogenesis through different pathogenetic pathways such as: mutation, cytotoxicity and enzymatic changes. A number of mechanisms have been proposed for these pathways including; DNA damage, labialization of lysosomes and calcium mediated toxicity (6). The emphasis on teratogenic influences has not led to elucidation of pathogenetic pathways, so the potential mechanisms of induction of cleft palate defects by SM are considered to be important areas of research in future.

In a similar study done by McNamara et al. (33) pregnant rats were exposed to SM by gastric intubation in different doses. It was claimed that, no evidence of teratogenicity was observed. Such a discrepancy results could be explained by different routes of drug administration and doses which had been used.

This study indicated that within a population of pregnant mice, exposure to SM was directly correlated with increased risk of congenital cleft malformations. Therefore, the transplacentally exposed fetus which may survive SM attack can bear long term consequences. Our data demonstrate that the teratogenic effects of SM are restrictively under the influence of the time of gestation (during organogenesis), as well as the threshold dose. Considering the destructive effects of mustard gas on different organs, the logical question that one may ask is why despite the conventional laws that prohibit the use of these weapons, nevertheless, it has been recently used against the innocent human beings. 
